# Horizontal transfer of OC1 transposons in the Tasmanian devil

**DOI:** 10.1186/1471-2164-14-134

**Published:** 2013-02-27

**Authors:** Clement Gilbert, Paul Waters, Cedric Feschotte, Sarah Schaack

**Affiliations:** 1 Université de Poitiers, UMR CNRS 7267, Ecologie et Biologie des Interactions, Equipe Ecologie Evolution Symbiose, Poitiers, France; 2 Evolution Ecology and Genetics, Research School of Biology, The Australian National University, Canberra, Australia; 3 School of Biotechnology & Biomolecular Sciences, Faculty of Science, University of New South Wales, Sydney, Australia; 4 Department of Human Genetics, University of Utah School of Medicine, Salt Lake City, UT, USA; 5 Department of Biology, Reed College, Portland, OR, USA

**Keywords:** Horizontal transfer, Transposable element, Tasmanian devil, *Sarcophilus harrisii*

## Abstract

**Background:**

There is growing recognition that horizontal DNA transfer, a process known to be common in prokaryotes, is also a significant source of genomic variation in eukaryotes. Horizontal transfer of transposable elements (HTT) may be especially prevalent in eukaryotes given the inherent mobility, widespread occurrence, and prolific abundance of these elements in many eukaryotic genomes.

**Results:**

Here, we provide evidence for a new case of HTT of the transposon family *OposCharlie1* (OC1) in the Tasmanian devil, *Sarcophilus harrisii.* Bioinformatic analyses of OC1 sequences in the Tasmanian devil genome suggest that this transposon infiltrated the common ancestor of the Dasyuridae family ~17 million years ago. This estimate is corroborated by a PCR-based screen for the presence/absence of this family in Tasmanian devils and closely-related species.

**Conclusions:**

This case of HTT is the first to be reported in dasyurids. It brings the number of animal lineages independently invaded by OC1 to 12, and adds a fourth continent to the pandemic-like pattern of invasion of this transposon. In the context of these data, we discuss the evolutionary history of this transposon family and its potential impact on the diversification of marsupials.

## Background

Much like horizontal gene transfer (HGT), the frequency, mechanisms and evolutionary impact of the horizontal transfer of transposable elements (HTT) have been well characterized in prokaryotes [1,2]. In eukaryotes, the number of fully sequenced genomes is now sufficient to reveal that HTT has also had a significant impact on the evolution of genomes in this group, but many aspects of the phenomenon remain largely mysterious [3,4]. Among metazoans, and in particular vertebrates, HTT can be examined on a geological timescale because these taxa are characterized by large, slow-evolving genomes in which vast numbers of transposable elements (TEs) can persist for many millions of years. In a series of recent studies, we and others reported numerous cases of Class II TEs (cut-and-paste DNA transposons) transferred horizontally among vertebrate and invertebrate species, between multiple continents, over a period stretching from ~45 to ~15 My ago [5-8]. These HTT events are notable because they are geographically widespread, implicate several transposon families, and affect a wide range of tetrapods, as well as a blood-sucking parasitic insect (*Rhodnius prolixus*) as a possible vector [6]. One of the transposon families, *OposCharlie1* (OC1), achieved a particularly high level of promiscuity, as it was found to have infiltrated the genomes of 11 deeply diverged species on both Old and New World continents at the time of transfer.

## Results and discussion

Sequence homology-based searches (BLASTn) using the OC1_Et consensus sequence as a query (reconstructed in [6]) yielded many significant hits in the genome of the Tasmanian devil (*Sarcophilus harrisii*). Majority-rule consensus sequences were reconstructed for an autonomous element (2,571 bp) encoding a 602-amino acid transposase and for a major family of non-autonomous elements (192 bp) based on an alignment of 25 representative copies of each type (Additional file 1). We called this autonomous element family OC1_Das to reflect the fact that, in addition to the Tasmanian devil, it is present in several other members of the family Dasyuridae (see below). In addition, non-autonomous partners of OC1_Das are called OC1_Das_NA. They form a high copy number, homogenous family of non-autonomous elements (also called miniature terminal inverted-repeat transposable elements, or MITEs), which likely result from amplification of an internally-deleted autonomous copy (deletion breakpoints occur at position 36 and 2337 of the consensus OC1_Das), a process typical of Class II transposons [9]. Further sequence mining using BLASTn and RepeatMasker [10] revealed that the Tasmanian devil’s genome contains 5,208 copies (or fragments of copies longer than 100 bp) of OC1_Das; of these, 387 are full length or slightly truncated autonomous elements and the rest correspond to short non-autonomous elements. Two studies recently produced whole genome sequence (WGS) data for several Tasmanian devil individuals [11,12]. The results presented here are based on analyses of the WGS produced by Miller et al. [11] only, but analyses (not shown) carried out on the other WGS data [12] yield very similar numbers.

An alignment of the OC1_Das autonomous consensus sequence with those reconstructed previously for vertebrate and invertebrate taxa known to harbor OC1 [6] revealed a very high level of nucleotide identity across the entire length of the elements (90 to 95%). Phylogenetic analyses of 30 randomly-selected copies of OC1_Das yielded a star topology and a lack of subfamily structure, indicating that OC1_Das amplified through a single major burst of transposition, followed by the accumulation of private mutations in its copies. This pattern is consistent with a scenario of neutral evolution after insertion in the genome (Additional file 2: Figure S1). In further support for the neutral evolution of OC1_Das transposase sequences, we found that dN/dS values calculated between the consensus OC1_Das and each of the 30 copies examined were all above 0.5 (mean = 0.88; SD = 0.22). In addition, a codon-based Z-test revealed no evidence of purifying selection acting on OC1_Das transposase genes after their insertion in the Tasmanian devil genome (p-values > 0.05 for 27 pairwise comparisons and p-values between 0.027 and 0.042 for the three other pairwise comparisons). Thus, as concluded in previous studies [5,6], the level of sequence conservation between OC1_Das and OC1 elements previously identified in other species is incompatible with vertical inheritance from the most recent common ancestor (dating between 80 My and 500 My ago [13,14]. For example, the level of nucleotide identity (95%) between OC1_Das and OC1_Md from the opposum (the closest species to Dasyuridae known to harbor OC1) is significantly higher (88%) than that of RAG1, a gene known to evolve under strong functional constraint in vertebrates (dN/dS = 0.1 between Tasmanian devil and opossum RAG1 sequences; codon Z-test p-value = 0). Thus, the presence of OC1 in the Tasmanian devil’s genome is best explained by the horizontal transfer of this element into this lineage. This discovery brings the total number of animal lineages independently infiltrated by OC1 to twelve.

**Figure 1:**
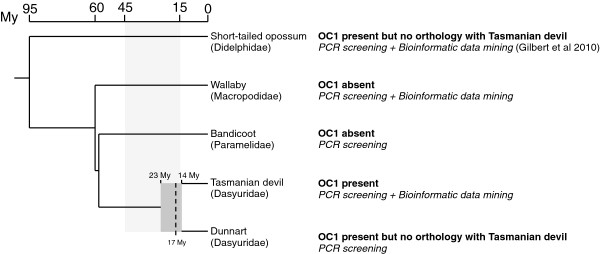
**Dated phylogeny summarizing the evidence for OC1_Das horizontal transfer and timing of activity in marsupials. **Phylogenetic relationships and divergence times between the five marsupials species (short-tailed opossum: *Monodelphis domestica*, wallaby: *Macropus eugenii*, eastern barred bandicoot: *Perameles gunnii*, Tasmanian devil: *Sarcophilus harrisii*, fat-tailed dunnart: *Sminthopsis crassicaudata*) are taken from Meredith et al. (2008), Meredith et al. (2009) and Krajewsky et al. (2000). The range of divergence times calculated for the split between the Tasmanian devil and the dunnart (23 to 14 My; dark grey rectangle) is illustrated to show that it overlaps with the timing of OC1_Das activity burst (dashed line at 17 My), derived from our molecular clock approach. The light grey rectangle illustrates the time range during which all OC1 HTs described so far in metazoans took place. See text for more details on our molecular dating of the OC1_Das burst of activity and PCR screening. Products obtained from the PCR screening were cloned and sequenced and are provided in Additional file 4 (122-bp fragments of non-autonomous OC1 in Tasmanian devil and fat-tailed dunnart) and Additional file 5, Additional file 6 and Additional file 7 (empty sites for three OC1_Das copies present in the Tasmanian devil but absent from the dunnart).

Next, we endeavored to estimate the age of invasion of Tasmanian devils by OC1 using two independent approaches. First, we estimated the timing of genomic amplification of OC1_Das, because a burst of transposition is most likely to occur quickly after HTT and, indeed, may be necessary for the element to successfully colonize a species [15]. To calculate this, we divided the average percent divergence between the consensus sequences and all copies of the TE in the genome (5.4%; Table 1) by an estimate of the neutral rate of nucleotide substitution (see Additional file 3: Figure S2 for the distribution of percent divergences). We are not aware of any estimate of the neutral rate of substitution for the Dasyuridae, and therefore used the rate we previously estimated based on genomic alignments of opossum and wallaby ancestral repeats (3.21 × 10^-9^, [5]). This yielded an approximate age estimate of 17 My ago (+/- 5.7 My) for the activity peak of OC1_Das, which falls within the range of the HTT events previously reported for this transposon (15 to 45 My ago [5,6]). As an independent assessment of the age of the OC1_Das invasion, we conducted a PCR screen of OC1 in several marsupial species with different divergence times from the Tasmanian devil. For this, we used a pair of internal OC1_Das primers designed from the consensus sequence to look for presence/absence of the element, as well as primer pairs flanking five individual insertions in the Tasmanian devil to look for presence/absence of the element at orthologous genomic positions in the other species. The results of this screen indicate that OC1_Das is undetectable in the tammar wallaby (*Macropus eugenii*) and bandicoot (*Perameles gunnii*) genomes (Figure 1). The absence of OC1_Das in wallaby was confirmed computationally by a BLASTn search of its draft whole genome sequence [16]. Our PCR screen detected OC1_Das in the dunnart (*Sminthopsis crassicaudata*, Additional file 4), but not at any of the three orthologous loci occupied in Tasmanian devil that we were able to successfully amplify. Sequencing of these ‘empty’ loci in the Dunnart genome and comparison with the occupied sites in Tasmanian devil revealed no evidence of transposon excision footprints (Additional file 5, Additional file 6 and Additional file 7), indicating that the absence of OC1_Das from these loci in the dunnart is most likely the result of lineage-specific insertions subsequent to the divergence of dunnart and Tasmanian devil.

**Table 1:** **Genome-wide characterization of OC1 elements based on WGS for various metazoans**^**1**^

	**Copy number**	**Amount of DNA (kb)**	**Average distance from consensus (%)**
**Tasmanian devil**	5208	1275	5.4
**Squirrel monkey**	>344	>64	4.1
**Tarsier**	519	278	4.0
**Mouse lemur**	12055	2278	8.5
**Bushbaby**	14687	3755	10
**Bat**	7190	1440	2.2
**Tenrec**	6837	1584	8.1
**Opossum**	6025	3098	10.5
**Lizard**	3902	2626	5.5
**Frog**	950	445	8.9
**Triatomine bug**	1286	495	6.4
**Planaria**	46	26	8.6

^1^Values based on [6] except for the Tasmanian devil.

Two scenarios can explain the patterns we observe. First, OC1_Das was horizontally transferred into a common ancestor of the dunnart and Tasmanian devil, and the absence of orthologous copies at the three loci that we tested is due to the fact that the element continued actively transposing after the split of the species, and/or to the differential fixation of ancestrally polymorphic insertions in the two species. The second possibility is that OC1 was transferred independently in the dunnart and Tasmanian devil lineages. Our molecular estimate of the timing of OC1_Das amplification (17 My) falls within the range of proposed divergence times for dunnart/Tasmanian devil (14 – 23 My; [14,17,18]), which supports the idea that OC1_Das was active during the speciation event that separated the Tasmanian devil from its dasyurid sister group that includes about 40 species from four genera (*Sminthopsis*, *Dasyurus*, *Antechinus* and *Phascogale*; [14,18]). Whether the amplification of OC1_Das played a role in this speciation event, as proposed for other transposition bursts [19], is an interesting question that deserves further investigation.

It is also noteworthy that we found one full-length OC1_Das transposase that is free of premature stop codons and frameshift mutations (contig AEFK01150217; [12]). While it is unclear whether this transposase is part of a complete autonomous element (it lies within a short 1975-bp contig), it may be a source of functional protein capable of mobilizing other OC1 copies. The question of whether this family of element is still active in Dasyuridae warrants further assessment, as to the best of our knowledge no active Class II transposon has yet been reported in marsupials.

**Figure 2:**
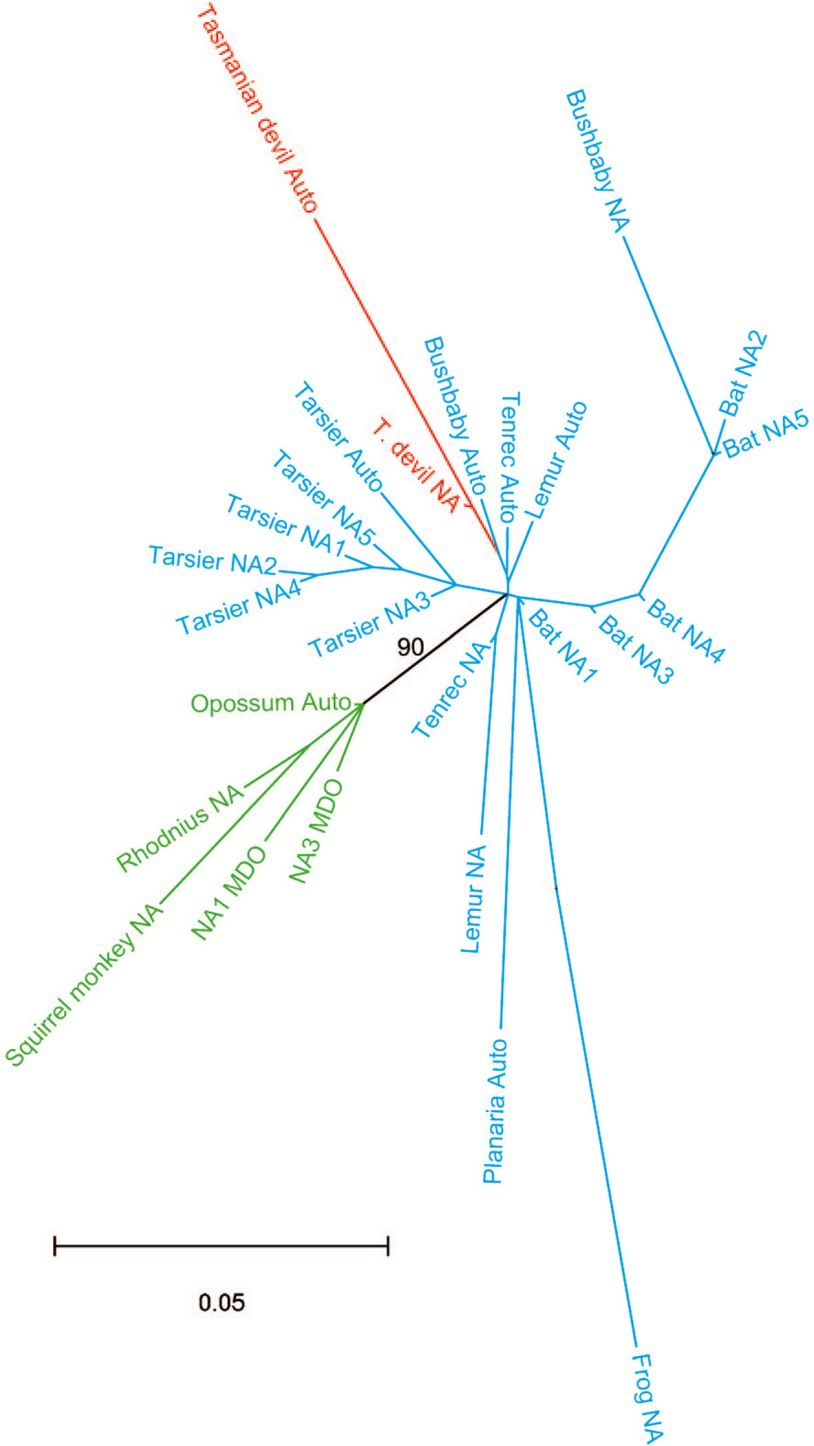
**Phylogenetic tree of all OC1 consensus sequences characterized to date.** The tree is based on an alignment of 3318 nucleotides. Only bootstrap values greater than 50 are shown. OC1 sequences other than OC1_Das are taken from Gilbert et al. (2010). Old World, New World, and Australian OC1 sequences are in blue, green and red, respectively. NA = non-autonomous OC1; Auto = Autonomous OC1.

With regard to the geographical location of HTT of OC1_Das, paleobiogeographical evidence strongly suggests that the Dasyuridae, as well as the ancestor of the dunnart + Tasmanian devil clade, originated in Australia, which is where the Tasmanian devil, dunnart, and most extant species of this family are still confined [14,18]. While the Tasmanian devil is only found in Tasmania, today its absence from mainland Australia is due to a recent extinction (<1000 years; [20]) and Tasmania was repeatedly connected to Australia during glacial periods through land bridges emerging within Bass Strait until 10,000 years ago [21]. Thus, we can confidently conclude that the horizontal transfer of OC1_Das took place in Australia, which adds a new continent to the previously reported transfers of this element in Asia, Africa and South America [6]. Our phylogenetic analysis places OC1_Das within the Old world OC1 clade, suggesting that the presence of this element in dasyurids is likely the result of a transcontinental movement of a vector species between Australasia and the Old World, rather than between Australiasia and the New world (Figure 2). We believe the lack of resolution in the topology of the OC1 phylogeny, both within (Additional file 2: Figure S1) and between species (Figure 2), reflects the fact that HTT events involving this element took place within a relatively short timeframe preventing sufficient time for accumulation of diagnostic mutations. Together these data further underscore the worldwide scale and pandemic-like pattern of these transposon invasions.

Several hypotheses have been proposed to explain how transposable elements might be transferred between eukaryotic hosts, including transmission by contact of bodily fluids, feeding, or via some kind of vector such as a parasite [22-24], reviewed in [3,25]. None of these routes can be favored at present in the case of the Dasyuridae. The location of OC1_Das in the Old World Clade of the OC1 phylogeny (Figure 2) does not help resolve this issue. However OC1 is an especially interesting case of HTT in that alignment of the 5′ subterminal region of the otherwise nearly identical consensus sequences from all taxa previously found to harbor horizontally-transferred copies of the element reveals two geographically distinct signatures (either Old or New World). Surprisingly, this subterminal region in OC1_Das is made of a 382-bp fragment that is unique among all OC1 elements so far described (Position 66 to 447 on the OC1_Das autonomous consensus provided in Additional file 1). Taxa that are found to share this region may provide evidence for concurrent HTT and open the possibility that this region of the transposon could be used to identify direct donor or vector species in the future.

## Conclusions

We provide evidence for a new case of HTT of the transposon family *OposCharlie1* (OC1) based on WGS of the Tasmanian devil, *Sarcophilus harrisii,* and molecular screening of closely-related marsupials*.* Our bioinformatic analysis and PCR results indicate the HTT event occurred ~17 My ago. This finding expands one of the most widespread case of HTT identified in eukaryotes to include another deeply diverged vertebrate lineage (the dasyurid marsupials) and an additional continent (Australia). Intriguingly, our estimated dating of this episode of HTT coincides with a series of speciation events that have led to the diversification of this lineage of marsupials.

## Methods

Searches for the presence of OposCharlie1 (OC1) in eukaryote genomes were performed using BLASTn and the OC1_Et consensus sequence reconstructed for the tenrec (*Echinops telfairi*) in [6]. Phylogenetic analyses of OC1 consensus sequences were reconstructed using PhyML 3.0 [26]. The best fitting nucleotide substitution model (TPM3uf + G) was chosen using jModeltest 0.1.1 [27]. The robustness of the nodes was assessed by a bootstrap analysis involving 1000 pseudo replicates of the original matrix. To examine the pattern of evolution of OC1 after horizontal transfer in the Tasmanian devil’s genome, dN/dS analyses were performed as follows: all non-sense mutations were removed from the transposase sequences of the 30 full (or nearly full) length copies aligned as described above and we then tested whether the dynamics of evolution between each copy and the consensus (an estimate of the ancestral founder element) was significantly different from what is expected if the sequence is evolving neutrally using the codon-based Z-test in MEGA 4.0 (Nei-Gojobori method; Jukes-Cantor correction; 1000 bootstrap replicates).

PCR of OC1_Das using internal primers and primers designed on flanking regions were conducted using the following temperature cycling: initial denaturation at 94°C for 5 min, followed by 30 cycles of denaturation at 94°C for 30 s, annealing between 48–54°C based on element-specific gradients (for 30 s), and elongation at 72°C for 1 min, ending with a 10 min elongation step at 72°C. Fragments from the PCR were visualized on a 1–2% agarose gel, cloned and sequenced.

## Competing interest

The authors declare that they have no competing interests.

## Authors’ contribution

CG participated in all aspects of the study. PW performed the molecular work, CF participated in the analysis and interpretation of the data, and SS conceived of and coordinated the study. All authors contributed to, read, and approved the final manuscript.

## Supplementary Material

Additional file 1 **A FASTA file containing the consensus sequence of OC1_Das autonomous (OC1_Das-Auto) and non-autonomous elements (OC1_Das-NA). **The transposase gene lies between position 565 and 2370 within OC1_Das-Auto. Click here for file

Additional file 2: Figure S1 Phylogenetic relationships of individual copies of OC1_Das. Thirty full (or nearly full) length autonomous elements were randomly selected and a multiple alignment was built using ClustalW in BioEdit 5.8 [28]. The name of the sequences corresponds to the GenBank accession number of the contig from which they were extracted, followed by the position within each contig of the OC1_Das sequence that was used in the analysis. The tree was constructed using the neighbor-joining method in MEGA 4.0 ([29]; maximum-likelihood composite model; 1,000 bootstrap pseudoreplicates). Bootstrap values are not shown because they are all lower than 50. Click here for file

Additional file 3: Figure S2 Distribution of percent divergence between each copy of OC1_Das and the consensus element. Click here for file

Additional file 4 **A FASTA file containing the OC1_Das-Auto and OC1_Das-NA consensus sequences aligned with two copies of OC1_Das-NA sequenced from the Tasmanian devil *Sarcophilus harrisii *(OC1_Das_NA-Sh1-2) and four copies of OC1_Das-NA sequenced from the dunnart, *Sminthopsis crassicaudata *(OC1_Das_NA-Sc1-4). **While positions 1–58 and 104–192 of OC1_Das_NA are homologous to the 5′ and 3′ region of OC1_Das-Auto, respectively, the internal region of OC1_Das_NA (positions 59–103) has an unknown origin. The internal region of OC1_Das_NA-Sc is also from an unknown origin and it is different from that of OC1_Das_NA-Sh. The sequence of the primers used to amplify OC1_Das_NA (melting temperature = 55 degrees) in both species is also included in the alignment (OC1_Das_NA_F/R-55). Click here for file

Additional file 5 **A FASTA file containing a copy of OC1_Das_NA extracted from the Tasmanian devil genome (contig GL962536) with its 5′ and 3′ flanking regions aligned with their homologous loci in *Macropus eugenii *(ABQO010294817), *Monodelphis domestica *(AAFR03026483) and *Sminthopsis crassicaudata *(empty site 1 sequenced in this study using primers F/R2536 with a PCR melting temperature of 53 degrees). **OC1_Das_NA is present in the Tasmanian devil (position 212–403 of contig GL962536) and is absent from the three other species. Click here for file

Additional file 6 **A FASTA file containing a copy of OC1_Das_NA extracted from the Tasmanian devil genome (contig GL972772) with its 5′ and 3′ flanking regions aligned with their homologous loci in *Macropus eugenii *(ABQO010316396), *Monodelphis domestica *(AAFR03014268) and *Sminthopsis crassicaudata *(empty site 2 sequenced in this study using primers F/R2772 with a PCR melting temperature of 55 degrees). **OC1_Das_NA is present in the Tasmanian devil (position 494–684 of contig GL972772) and is absent from the three other species. Click here for file

Additional file 7 **A FASTA file containing a copy of OC1_Das_NA extracted from the Tasmanian devil genome (contig GL964687) with its 5′ and 3′ flanking regions aligned with their homologous loci in *Macropus eugenii *(ABQO010134790) and *Sminthopsis crassicaudata *(empty site 2 sequenced in this study using primers F/R4687-55 with a PCR melting temperature of 55 degrees). **OC1_Das_NA is present in the Tasmanian devil (position 105–296 of contig GL964687) and is absent from the three other species. Click here for file
