# Supplementary figures and images for: Horizontal transfer of OC1 transposons in the Tasmanian devil
Source: BMC Genomics. 2013 Feb 27;14:134. doi: 10.1186/1471-2164-14-134 (PMC3621081; doi:10.1186/1471-2164-14-134)

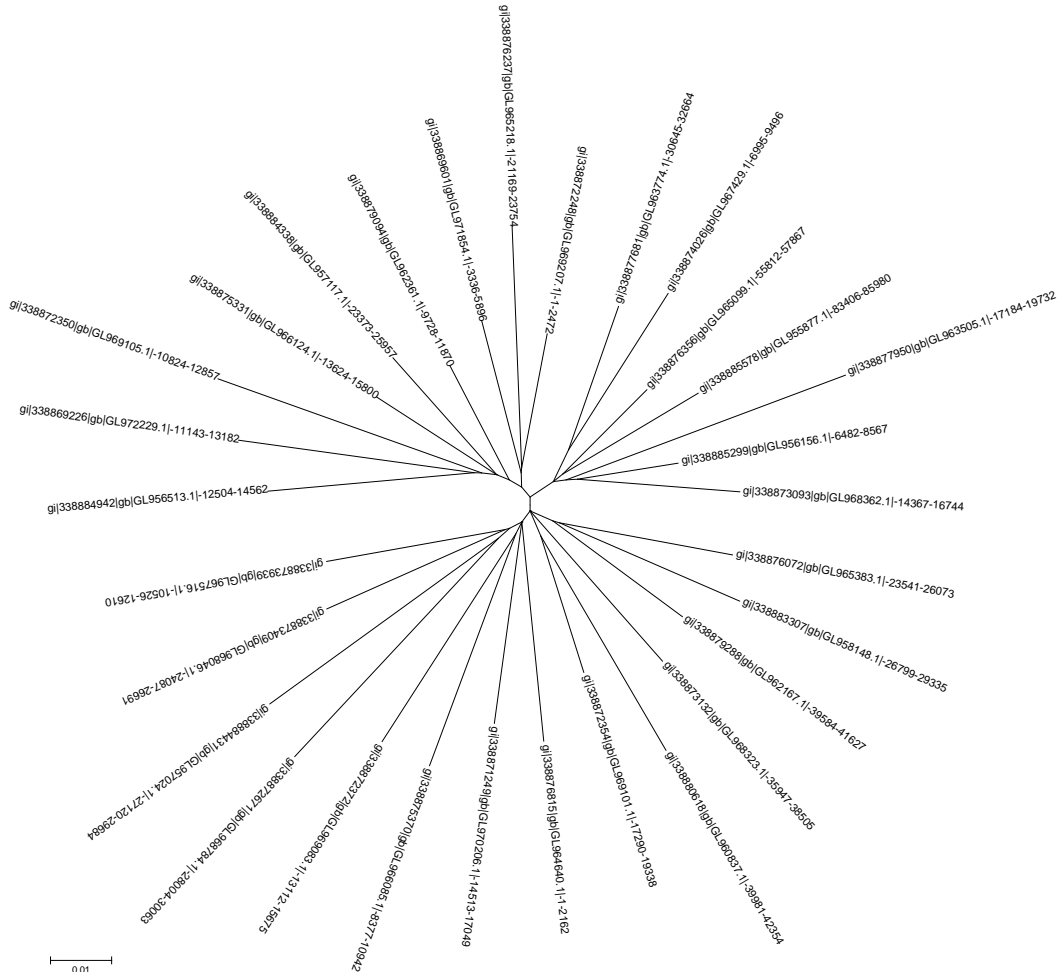

0.01

Supplement: Additional file 2: Figure S1 — Phylogenetic relationships of individual copies of OC1_Das. Thirty full (or nearly full) length autonomous elements were randomly selected and a multiple alignment was built using ClustalW in BioEdit 5.8 [28]. The name of the sequences corresponds to the GenBank accession number of the contig from which they were extracted, followed by the position within each contig of the OC1_Das sequence that was used in the analysis. The tree was constructed using the neighbor-joining method in MEGA 4.0 ([29]; maximum-likelihood composite model; 1,000 bootstrap pseudoreplicates). Bootstrap values are not shown because they are all lower than 50. [file 1471-2164-14-134-S2.pdf]

Number of  
copies

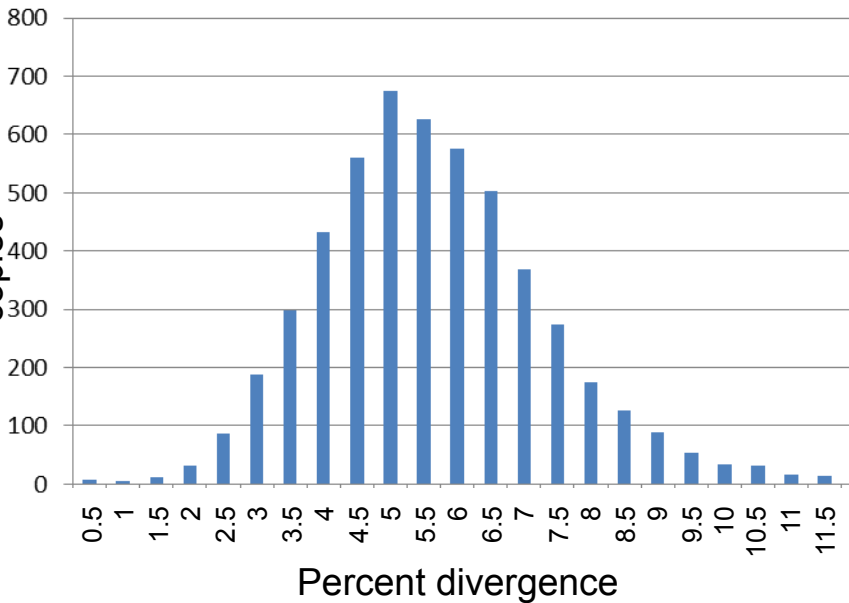

Supplement: Additional file 3: Figure S2 — Distribution of percent divergence between each copy of OC1_Das and the consensus element. [file 1471-2164-14-134-S3.pdf]
